# Impacts of extension access and cooperative membership on technology adoption and household welfare

**DOI:** 10.1016/j.jrurstud.2017.06.022

**Published:** 2017-08

**Authors:** Tesfamicheal Wossen, Tahirou Abdoulaye, Arega Alene, Mekbib G. Haile, Shiferaw Feleke, Adetunji Olanrewaju, Victor Manyong

**Affiliations:** a International Institute of Tropical Agriculture (IITA), Abuja, Nigeria; b International Institute of Tropical Agriculture (IITA), Ibadan, Nigeria; c International Institute of Tropical Agriculture (IITA), Lilongwe, Malawi; d Center for Development Research (ZEF), Bonn University, Germany; e International Institute of Tropical Agriculture (IITA), Dar es Salaam, Tanzania

**Keywords:** Impact evaluation, Extension access, Cooperatives, Adoption, Welfare, Nigeria

## Abstract

This paper examines the impacts of access to extension services and cooperative membership on technology adoption, asset ownership and poverty using household-level data from rural Nigeria. Using different matching techniques and endogenous switching regression approach, we find that both extension access and cooperative membership have a positive and statistically significant effect on technology adoption and household welfare. Moreover, we find that both extension access and cooperative membership have heterogeneous impacts. In particular, we find evidence of a positive selection as the average treatment effects of extension access and cooperative membership are higher for farmers with the highest propensity to access extension and cooperative services. The impact of extension services on poverty reduction and of cooperatives on technology adoption is significantly stronger for smallholders with access to formal credit than for those without access. This implies that expanding rural financial markets can maximize the potential positive impacts of extension and cooperative services on farmers’ productivity and welfare.

## Highlights

• Extension access and cooperative membership have a positive impact on technology adoption.
• Extension access and cooperative membership improves asset ownership and welfare.
• Extension access and cooperative membership have stronger effects for farmers with formal credit access.
• Expanding rural financial markets can maximize the potential positive impacts of extension and cooperative services.

## 1 Introduction

Adoption of improved agricultural technologies by smallholders is considered as the main pathway for breaking poverty trap. Applied correctly, adoption should, *ceteris paribus,* increase productivity and provide additional income to farmers. In this way, technology adoption can accelerate economic growth, create marketing opportunities, and help millions of farmers to move out of poverty. However, adoption rates for improved agricultural technologies have been rather disappointing and far from complete and proper identification of the main barriers of adoption remains a challenge (Shiferaw et al., 2008, Solomon et al., 2010, Wossen et al., 2015). Major identified causes of low adoption rates include supply-side constraints such as imperfect information and credit markets (Shiferaw et al., 2008, Suri, 2011, Wossen et al., 2015). Addressing information market imperfections can therefore serve as an important entry point for increasing adoption of agricultural technologies.

This paper focuses on extension access and cooperative membership which are key supply-side policy instruments to influence agricultural productivity in developing countries. Access to extension service enhances the adoption of improved agricultural technologies by reducing supply-side constraints that arise due to information market inefficiencies (Wossen et al., 2015). In particular, extension access facilitates adoption by exposing farmers to new technologies and by educating them about best farming and management practices (Anderson and Feder, 2007, Wossen et al., 2013). In addition to its direct effect on adoption, access to extension service affects welfare by helping farmers to reduce the gap between potential and actual yields (Anderson and Feder, 2007). However, extension access may also hinder adoption if extension workers exclude the poorest farmers or if they lack both the incentive and accountability needed to transfer reliable and timely information to smallholders (Davis, 2008, Jack, 2011). Although extension networks have been cited as the primary ways through which researchers and policymakers promote new and improved agricultural technologies, the evidence for their impact on adoption and welfare is rather mixed (Anderson and Feder, 2007, Davis et al., 2012).

The other well-documented constraint to the adoption of agricultural technologies is related to market inefficiencies, financial as well as input and output markets. In response to these market-related barriers, several farmer-controlled cooperatives have emerged in rural areas (Latynskiy and Berger, 2016). Cooperatives are widely regarded as an important institutional innovation that can help overcome the constraints that impede smallholders’ access to market (Abebaw and Haile, 2013, Verhofstadt and Maertens, 2014, Ma and Abdulai, 2016). There are many pathways through which cooperatives may affect technology adoption and welfare. First, cooperatives can relax the liquidity constraint that farmers face by providing credit for members. Secondly, cooperatives affect adoption and welfare by providing market information and, thirdly, by potentially offering a better market price for their produce. Finally, by pooling different resources such as credit, information, and labour among members, cooperatives can create economies of scale and hence improve welfare.

Against this backdrop, this paper seeks to examine the impact of extension access and cooperative membership not only on adoption of improved agricultural technologies but also on household welfare outcomes. Whereas adoption of improved cassava varieties serves as a measure of the adoption of agricultural technology, we use asset ownership, consumption expenditure, and the progress out of poverty index (PPI) as a measure of welfare outcomes. Evaluating the impact of extension access and cooperative membership on technology adoption and welfare outcomes is nontrivial as in other social programs, because of endogenous program placements. We therefore employed alternative econometric techniques including propensity score matching and endogenous switching regression methods to address the endogeneity bias problem. By focusing on a country that heavily relies on cassava, this paper uses empirical data to identify *the causal effects of access to extension and cooperative services on the adoption of improved cassava varieties and household welfare.* In doing so, the study provides not only new evidence on the impacts of extension access and cooperative membership on welfare outcomes but also on the heterogeneous treatment effects of such interventions. To the authors' knowledge, this paper is the first to provide a comprehensive assessment of extension access and cooperative membership effects on adoption and welfare outcomes in the context of Nigeria.

The remainder of the paper is organized as follows: Section 2 provides background information on the evolution of extension services and cooperatives and reviews the literature on the impacts of extension access and cooperative membership on technology adoption and household welfare. Section 3 presents data sources and the econometric strategy used for the empirical analysis. Section 4 presents the findings and discusses the results. Section 5 concludes the study, provides a list of open questions and discusses further research.

## 2 Context and related literature

Our study focuses on cassava production in Nigeria, the largest cassava producer in the world. Cassava is the most widely cultivated root crop in terms of area allocation and has the largest number of growers (FMANR (Federal Ministry of Agriculture and Natural Resources), 2010, Abdoulaye et al., 2013). Cassava has been increasing in importance in recent years and is fast replacing yam and other traditional staple foods as a famine reserve and insurance crop against hunger (FAO and IFAD, 2005, FMANR (Federal Ministry of Agriculture and Natural Resources), 2010). The crop is important not only as a food but also as a major source of income for rural households. As a cash crop, cassava generates income for the largest number of households compared with other staples (FAO and IFAD, 2005, FMANR (Federal Ministry of Agriculture and Natural Resources), 2010); this justifies our focus on the crop. Improving agricultural productivity - in particular, cassava productivity - through an efficient extension advisory service is therefore central for poverty reduction efforts in Nigeria. Cognizant of this fact, different approaches and systems for extension service delivery have been implemented in an attempt to improve productivity and reduce rural poverty. For instance, until the late 1960s the extension service mainly targeted exportable commodities. However, this approach was reversed in the 1980s when the focus shifted towards food self-sufficiency as part of the Agricultural Development Projects (ADPs) program. This approach gave special attention to training and visit (T&V) that was favoured by many donors including the World Bank (FMARD, 2014). The services provided by ADPs include establishing demonstration farms; identifying lead farmers and providing them with information about improved farming practices; facilitating access to improved technology and inputs, such as improved seed varieties, fertilizer, crop chemicals and machinery services and helping lead farmers to train other farmers (Mogues et al., 2012). To date, T&V is still the dominant extension service delivery approach in Nigeria, albeit with some modifications and blending with participatory approaches by several NGOs in the agricultural sector (FMARD, 2014).

With the aim of improving the effectiveness and efficiency of the extension service, a policy of a Unified Agricultural Extension Service (UAES) was implemented in 1991. This program aimed at providing an 'efficient extension service through a single extension agent covering the whole farming system in a holistic manner. From 2008 onwards, the Government again revised the extension policy as part of the National Food Security Program (NFSP) to further improve efficiency through information and interventions based on communication technology (ICT) (FMARD, 2014). The new extension system was aimed at transforming agricultural extension service into a participatory, demand-driven, market-oriented, and ICT-driven service (FMARD, 2014).

In the context of sub-Saharan Africa (SSA), the role that extension access plays in technology transfer and household welfare has received considerable attention (Anderson and Feder, 2007, Davis, 2008, Davis et al., 2012). Empirical evidence has shown that institutional arrangements and public investment that improve agricultural extension play a crucial role in facilitating technology transfer for rural poor farmers (Anderson and Feder, 2007, Davis, 2008, Dercon et al., 2009, Ito et al., 2012). For instance, Owens et al. (2003) reported a 15% gain in crop productivity due to extension access in rural Zimbabwe. Similarly, Dercon et al. (2009) showed that agricultural extension improved household welfare by reducing the incidence of poverty in rural Ethiopia. Their study highlighted the fact that receiving at least one extension visit reduced the incidence of poverty by 10 percentage points and increased consumption growth by 7 percentage points. In the context of Nigeria, Abdoulaye et al. (2013) found that farmers’ proximity to change agents resulted in a higher level of awareness and the use of improved technologies. Similarly, Sodiya et al. (2007) reported a positive relationship between extension access and adoption of improved cassava varieties in Nigeria. Despite a plethora of empirical evidence on the effects of extension access in many developing countries, a comprehensive assessment of the effects of extension access on technology adoption and household welfare is almost non-existent in the context of Nigeria.

Another important area of research in SSA is the role that rural producer organizations and agricultural cooperatives play for technology adoption and household welfare. In Nigeria, the history of cooperatives goes back to 1926 when the cocoa farmers formed a small union to sell their production. They emerged as a self-help group in response to local-level credit market imperfections. Rural producer organizations and cooperatives have long been studied in relation to transaction costs and collective action problems (Gabre-Madhin, 2001, Shiferaw et al., 2008, Bernard, 2009, Bernard and Taffesse, 2012, Ragasa and Golan, 2014), with particular interest in its application in developing countries (Ito et al., 2012, Abebaw and Haile, 2013, Latynskiy and Berger, 2016). Empirical evidence from many developing countries shows that agricultural cooperatives play a crucial role for technology adoption in the presence of high transaction costs and low bargaining power (Abebaw and Haile, 2013, Abdoulaye et al., 2013, Ma and Abdulai, 2016). In the context of SSA, cooperatives have been widely discussed in terms of improving market bargaining power (Shiferaw and Hellin, 2011, Abdoulaye et al., 2013, Latynskiy and Berger, 2016) and in facilitating risk-sharing (Shiferaw et al., 2008). In a study that assessed the role of cooperative membership on farmers' uptake of innovations, Kolade and Harpha (2014) eported that cooperative membership exerts a direct influence on adoption of innovations. Similarly, Gebremichael (2014) found that cooperative membership improves technology adoption and food security, especially for rural women. Their result suggests that cooperative membership helps households to diversify their livelihoods, provides opportunities to reduce transactional costs, improves market bargaining power and promotes opportunities for gender equity. In the context of Ethiopia, Abebaw and Haile (2013) reported a strong positive impact of cooperative membership on fertilizer adoption. Similarly, in their analysis of cooperative membership in rural China, Ma and Abdulai (2016) found a positive and significant impact of cooperative membership on apple yields, net returns, and household income.

However, cooperatives may impose costs on poor members in the form of compulsory regular membership fees. This is particularly the situation when farmers join cooperatives owing to social pressure instead of pure economic benefits. In addition, cooperatives may exclude disadvantaged groups and the poorest farmers. While it is increasingly clear that informal cooperatives can affect adoption decisions and welfare levels, it is likely to be heavily contingent on the specific technology, the type of resources required for getting access to it, and the socio-economic characteristics of members (Ito et al., 2012, Abebaw and Haile, 2013, Verhofstadt and Maertens, 2014, Latynskiy and Berger, 2016, Ma and Abdulai, 2016).

## 3 Data and methods

### 3.1 Data source and outcome indicators

The data for this study were obtained from the Cassava Monitoring Survey for Nigeria (CMS), which was designed to assess the adoption of improved cassava cultivars. The data were collected through a household survey from 2500 randomly selected cassava-growing households in the major cassava-growing States that together account for over 80% of the annual total production. The survey contains detailed information on a range of socio-economic attributes, asset holdings, poverty measures, adoption of improved cassava varieties, membership of formal and informal associations as well as access to credit and extension, among others. Since the focus is on welfare impacts of cooperative membership and extension access, we used PPI, asset ownership and consumption expenditure as a measure of welfare.

Our first welfare measure-PPI is introduced by the Grameen foundation with the specific aim of measuring poverty at the household level based on observable household characteristics, asset ownership and access to basic services (Desiere et al., 2015). PPI is constructed based on ten questions that are correlated with poverty and is widely used by many projects in many developing countries.^1^ The PPI indicator for Nigeria is based on data from the 2003/4 National Living Standards Survey (NLSS). Each question has a weight that helps to identify the likelihood that the household is living below the poverty line. All points in the scorecard are non-negative integers and the total score ranges from 0 (most likely to be below a poverty line) to 100 (least likely to be below a poverty line).

Our second welfare indicator is asset ownership. The use of asset ownership as a welfare indicator has two advantages. First, as argued by Carter and Barrett (2006), asset-based outcome indicators are forward-looking unlike indicators based on expenditure and income (i.e., asset based indicators show whether households are likely to remain poor into the future). As such, the use of asset ownership indicators enables us to address questions surrounding households’ longer-term prospects of being non-poor (Carter and Barrett, 2006). We therefore use the monetary value of productive assets (such as farm equipment), household assets (such as ownership of mobile phones and television sets) and durable assets (such as jewellery and household utensils) as a measure of asset value. Secondly, our asset-based outcome indicator can be used as a robustness check for our first preferred indicator, PPI score. Because PPI score accounts for household assets, we expect extension access and cooperative membership to have similar effects on PPI and asset ownership. The third outcome indicator we employ in this study is per-capita food consumption expenditure. Our intermediary outcome indicator, that is, adoption of improved cassava varieties, is measured by a dummy variable which takes a value of one if a household grows improved cassava varieties, and zero otherwise.

### 3.2 Empirical strategy

Identification of the causal effects of extension access and cooperative membership on potential outcome indicators is not trivial due to endogeneity bias. Accurate measurement of impacts requires controlling for both observable and unobservable characteristics through random assignment of individuals into treatments. In the absence of random assignments, selection bias may persist as observed and unobserved characteristics of individuals may affect the likelihood of receiving treatments as well as outcome indicators. In this paper, we employ propensity score matching (PSM), inverse probability weighted adjusted regression (IPWRA) and endogenous switching regression (ESR) approaches to control for endogeneity bias. The basic idea behind PSM is to match each treated household with a similar untreated household and then measure the average difference in the outcome variable between the treated and untreated households. In other words, we are interested in the question, “*How would the welfare level of households have changed had the treated households chosen not to be in the treatment group*?” Following Imbens and Wooldridge (2009), the average treatment effect on the treated (ATT) is defined as:

(1) ATT=E[Y(1)−Y(0)|T=1]

where Y(1) and Y(0) are outcome indicators (in our case, welfare and adoption level of treated and untreated households, respectively). T is a treatment indicator. However, we can only observe E[Y(1)|T=1] in our data set and E[Y(0)|T=1] is missing. In essence, we cannot observe the welfare and adoption level of treated households had they not been treated, once they are treated. Simple comparison of adoption and welfare level of farmers with and without treatment status introduces bias in estimated impacts due to self-selection bias. The magnitude of self-selection bias is formally presented as:

(2) E[Y(1)−Y(0)|T=1]=ATT+E[Y(0)|T=1−Y(0)|T=0]

By creating comparable counterfactual households for treated households, PSM reduces the bias due to observables. Once households are matched with observables, PSM assumes that there are no systematic differences in unobservable characteristics between treated and untreated households. Given this assumption of conditional independence and the overlap conditions, ATT is computed as follows:

(3) ATT=E[Y(1)|T=1,p(x)]−E[Y(0)|T=0,p(x)]

However, ATT from PSM can still produce biased results in the presence of mis-specification in the propensity score model (Robins et al., 2007, Wooldridge, 2007, Wooldridge, 2010). A potential remedy for such misspecification bias is to use IPWRA. According to Wooldridge (2010), IPWRA estimates will be consistent in the presence of mis-specification in the treatment/outcome model, but not both. As a result, the IPWRA estimator has the double-robust property that ensures consistent results as it allows the outcome and the treatment model to account for mis-specification. Following Imbens and Wooldridge (2009), ATT in the IPWRA model is estimated in two steps. Suppose that the outcome model is represented by a linear regression function of the form $Y_{i}=\alpha_{i}+\varphi_{i}x_{i}+\varepsilon_{i}$ for i=[01] and the propensity scores are given by p(x;γ). In the first step, we estimate the propensity scores as $p\left(x;\hat{\gamma }\right).$ In the second step, we then employ linear regression to estimate $\left(\alpha_{0},\;\varphi_{0}\right)$ and $\left(\alpha_{1},\;\varphi_{1}\right)\;$ using inverse probability weighted least squares as

(4) $$\underset{\alpha_{0},\;\varphi_{0}}{\min}\sum_{i}^{N}\left(Y_{i}-\alpha_{0}-\varphi_{0}x_{i}\right)/p\left(x,\hat{\gamma }\right)\;if\;T_{i}=0$$

(5) $$\underset{\alpha_{1},\;\varphi_{1}}{\min}\sum_{i}^{N}\left(Y_{i}-\alpha_{1}-\varphi_{1}x_{i}\right)/p\left(x,\hat{\gamma }\right)\;\;if\;\;T_{i}=1$$

The ATT is then computed as the difference between Eq. (4) and Eq. (5).

(6) $$ATT=\frac{1}{{\text{N}}_{\text{w}}}\sum_{i}^{N_{w}}\left[\left({\hat{\alpha }}_{1}-{\hat{\alpha }}_{0}\right)-\left({\hat{\varphi }}_{1}-{\hat{\varphi }}_{0}\right)x_{i}\;\right]\;$$

where, $\left({\hat{\alpha }}_{1},{\hat{\varphi }}_{1}\right)$ are estimated inverse probability weighted parameters for treated households while $\left({\hat{\alpha }}_{0},{\hat{\varphi }}_{0}\right)$ are estimated inverse probability weighted parameters for untreated households. Finally, ${\text{N}}_{\text{w}}$ stands for the total number of treated households.

However, matching techniques-regardless of adjustments for misspecification bias-can overcome only the selection bias caused by observables. When the cause of endogeneity bias is unobservable heterogeneity, such as farmer's inherent skill, results based on matching techniques will be biased. We, therefore, employed an ESR model that accounts for both observed and unobserved sources of bias (Lokshin and Sajaia, 2004, Shiferaw et al., 2014, Ma and Abdulai, 2016). The ESR approach addresses this endogeneity problem by estimating the selection and outcome equations simultaneously using the full information maximum likelihood (FIML) (Lokshin and Sajaia, 2004, Ma and Abdulai, 2016). We assume that a particular farm household would consider receiving a treatment if the expected benefit of the treatment (in terms of welfare gain) is positive. Let $w_{0}$ be the welfare level of households without treatment (that is, without extension access or cooperative membership), and let $w_{1}$ be the corresponding welfare level with treatment (with extension access or cooperative membership). The farmer will choose to be in the treatment, if the welfare gain defined as, $Y_{i}^{*}=w_{1}-w_{0}\text{,}$ is positive. However, the welfare gain that the farmer derives from treatment $\left(Y_{i}^{*}\right)$ is a latent variable determined by observed characteristics $\left(Z_{i}\right)$ as follows:

(7) $$\;Y_{i}^{\text{*}}=\beta_{0}+\gamma Z_{i}+\mu_{i}\;\text{with}\;T_{i}=\{\begin{array}{ccc} 1 & if & Y_{i}^{\text{*}}>0\\ 0 & if & Y_{i}^{\text{*}}\leq 0 \end{array}$$

The vector Z represents variables that affect the expected benefits from having extension access and being a member of cooperatives. The outcome function conditional on treatment can then be specified as ESR model in the following manner.

(8) $$Regime1:\;Y_{1i}=\gamma_{1}x_{1i}+\;\varepsilon_{1i}\;\;\text{if }\;\;T_{i}=1$$

(9) $$Regime\;2:\;Y_{2i}=\gamma_{2}x_{2i}+\varepsilon_{2i}\;\text{if }\;T_{i}=0$$

where, $Y_{1i}$ represents the outcome indicator of treated households and $Y_{2i}$ of untreated households while $x_{i}$ represents a vector of exogenous variables. $\;\varepsilon_{i}$ is the error term of the outcome variable. The error terms in the selection Eq. (7) and the outcome Eqs. (8), (9) are assumed to have a trivariate normal distribution with mean zero and covariance matrix (Ω) in the following manner:

$$\Omega =\;\left[\begin{array}{ccc} \sigma_{u}^{2} & \sigma_{1\mu } & \sigma_{2\mu }\\ \sigma_{1\mu } & \sigma_{1}^{2} & .\\ \sigma_{2\mu } & . & \sigma_{2}^{2} \end{array}\right]$$

where $\sigma_{u}^{2}=\mathrm{var}\left(\mu_{i}\right)\text{,}$ $\sigma_{1}^{2}=var\left(\;\varepsilon_{1}\right)\text{,}$ $\sigma_{2}^{2}=var\left(\;\varepsilon_{2}\right)\text{,}$ $\sigma_{1\mu }=cov\left(\mu_{i},\;\;\varepsilon_{1}\right)\text{,}$ $\sigma_{2\mu }=cov\left(\mu_{i},\;\;\varepsilon_{2}\right)$ Furthermore, $\sigma_{u}^{2}$ is estimable up to a scale factor and can be assumed to be equal to 1 (Maddalla, 1983) and $cov\left(\;\varepsilon_{1},\;\;\varepsilon_{2}\right)$ is not defined as $Y_{1}$ and $Y_{2}$ cannot be observed simultaneously. Moreover, the correlation between the error term of the selection equation and the outcome equation is not zero (i.e., $corr\left(\mu_{i},\;\;\varepsilon_{1}\right)\neq 0$ & $\;corr\left(\mu_{i},\;\;\varepsilon_{2}\right)\neq 0$) which creates selection bias. ESR addresses this selection bias by estimating the inverse mills ratios $\left({\text{λ}}_{1i}\;\text{and}\;{\text{λ}}_{2i}\right)$ and the covariance terms $\left(\sigma_{1\mu }\;\text{and}\;\sigma_{2\mu }\right)$ and including them as auxiliary regressors in Eqs. (8), (9). If $\sigma_{1\mu }$ and $\sigma_{2\mu }$ are significant, we reject the absence of selection bias. In addition, $\sigma_{1\mu }<0$ represents positive selection bias (i.e., households with above-average outcomes are more likely to choose to be in the treatment). The ESR model estimates can then be used to estimate ATT and ATU (Average treatment effect on untreated households) as follows:

(10) $$E\left(Y_{1i}\text{|}\;T_{i}=1\right)=\gamma_{1}x_{1i}+{\text{ λ}}_{1i}\sigma_{1\mu }$$

(11) $$E\left(Y_{2i}\text{|}\;T_{i}=0\right)=\gamma_{2}x_{2i}+{\text{ λ}}_{2i}\sigma_{2\mu }$$

(12) $$E\left(Y_{2i}\text{| }T_{i}=1\right)=\gamma_{2}x_{1i}+{\text{ λ}}_{1i}\sigma_{2\mu }$$

(13) $$E\left(Y_{1i}\text{| }T_{i}=0\right)=\gamma_{1}x_{2i}+{\text{ λ}}_{2i}\sigma_{1\mu }$$

The ATT is then defined as the difference between Eqs. (10), (12).^2^

(14) $$\begin{array}{l} \text{ATT}=E\left(Y_{1i}\text{|}\;T_{i}=1\right)-E\left(Y_{2i}\text{| }T_{i}=1\right)\\ =x_{1i}\left(\gamma_{1}-\gamma_{2}\right)+{\text{ λ}}_{1i}\left(\sigma_{1\mu }-\sigma_{2\mu }\right) \end{array}$$

Identification of the ESR model requires at least one additional variable as an instrument in Eq. (7) (that is, a variable that is correlated with the treatment status but not with outcome indicators). The challenge in our identification strategy is therefore to find a variable that directly affects the choice into treatment but not the outcome indicators. Following the literature (see, Alene and Manyong, 2007, Shiferaw et al., 2014), we used an indicator variable for the *presence of private local cassava processors* as a potential instrument for cooperative membership and village-level variables for extension access. In particular, we used village-level penetration rates of mobile phone coverage as an identifying instrument. Village-level mobile phone coverage is arguably beyond the households’ own decision.

### 3.3 Descriptive statistics

Table 1 presents the descriptive statistics of the key variables of interest. The data show that about 60% of farmers have adopted improved cassava varieties. However, the intensity of adoption is only 38%. The average household size is about 4.5 members and the average household head is 51 years old (Table 1). Moreover, most of the respondents are literate, with an average of 9 years of schooling for the household head. About 89% of household heads are male and 88% are married. Smallholder farmers are located on average 12 km away from a nearby fertilizer dealer and 11.7 km away from herbicide dealers. In terms of the treatment variables, the data show that about 24.5% of farm households are cooperative members while 39% are reported to have access to extension.

**Table 1:** Descriptive statistics by treatment.

Variable	1	2	3	4	5	Mean diff	Mean diff
	Total sample (N = 2190)^a^	With extension (N = 920)	Without extension (N = 1490)	Members of cooperatives (N = 602)	Non-members (N = 1808)	(2–3)	(4–5)
		Mean	Mean	Mean	Mean		
PPI Index	59.9	65.3	56.5	64.9	58.3	8.8^∗∗∗^	6.7^∗∗∗^
Log of asset value	4.77	5.04	4.6	5.18	4.64	0.44^∗∗∗^	0.54^∗∗∗^
Log per-capita food expenditure	11.62	11.66	11.59	11.64	11.62	0.066^∗^	0.02
Adoption	0.597	0.74	0.51	0.71	0.56	0.23^∗∗∗^	0.15^∗∗∗^
Household size (Family size in numbers)	4.5	4.69	4.38	4.8	4.4	0.31^∗∗∗^	0.4^∗∗∗^
Age (Age of the household head in years)	51	50.7	51.2	51.22	50.92	−0.5	0.3
Sex of the household head (1 = male)	0.89	0.92	0.87	0.89	0.88	0.05^∗∗∗^	0.01
Marital status (1 = married)	0.88	0.92	0.86	0.90	0.88	0.06^∗∗∗^	0.02
Education (years of schooling)	8.8	9.4	8.4	9.9	8.4	1.00^∗∗∗^	1.5^∗∗∗^
Total farm size (ha)	2.93	3.45	2.62	3.34	2.8	0.83^∗∗∗^	0.54^∗∗∗^
Livestock ownership (TLU^b^)	0.60	0.923	0.407	1.43	0.33	0.516	1.1^∗∗^
Access to credit (1 = yes, 0 = otherwise)	0.434	0.54	0.37	0.61	0.37	0.17^∗∗∗^	0.24^∗∗∗^
Use of chemical fertilizer	0.36	0.32	0.38	0.362	0.359	−0.06^∗∗∗^	0.003
Distance from fertilizer dealer (km)	11.98	12.1	11.9	11.75	12.1	0.2	−0.26
Distance from herbicide dealer (km)	11.68	11.21	11.98	11.6	11.7	−0.77	−0.1
Presence of private processors (1 = yes, 0 = otherwise)	0.17	0.258	0.111	0.299	0.124	0.147^∗∗∗^	0.174^∗∗∗^
Mobile phone coverage (1 = yes, 0 = otherwise)	0.88	0.87	0.89	0.89	0.88	−0.19	0.01

a The total sample size in the study was about 2500. However, our final sample is 2190 due to missing values for expenditure and other controls.

b Note: TLU = Tropical Livestock Unit.

Table 1 further presents the difference in means (of all covariates) between farmers with and without extension access as well as between cooperative members and non-members. The mean differences are statistically significant for all our outcome indicators. For instance, about 74% of households with extension access adopted improved cassava varieties while only 51% of farmers without extension access adopted improved cassava varieties. The difference in the mean adoption rate between the two groups is statistically significant at 1%. On average, households with extension access own more land, have better credit and market access and tend to be wealthier than those without access.

Surprisingly, farmers with access to extension tend to use less chemical fertilizer than households without extension access. Similarly, about 72% of cooperative members and only 56% of non-members adopted improved cassava varieties. This difference is also statistically significant at 1%. In addition, cooperative members tend to be better educated, have larger household size, farm size, and more assets (measured by ownership of livestock wealth), and have better credit access. Farm households with extension access have significantly higher asset values and PPI scores than those without extension access.

Similarly, cooperative members have better asset values and PPI scores compared to non-members. However, as stated earlier, the results in Table 1 cannot be used to make inferences regarding the impacts of access to extension services and cooperative membership on poverty and welfare or technology adoption without controlling for other confounding factors.

## 4 Results

### 4.1 Determinants of extension access and cooperative membership

Table 2 presents the main determinants of extension access and cooperative membership.^3^ The results show that extension access is strongly associated with the socio-economic and demographic characteristics of households. In particular, older and more educated households are more likely to seek extension services. Farm size has a negative and statistically significant effect on the probability of receiving extension services, implying that extension agents are more likely to target smallholders. Similarly, households with access to formal credit are more likely to seek extension services. Regarding the determinants of cooperative membership, farm households with larger land sizes and more livestock units are more likely to become cooperative members. Education has a positive and statistically significant coefficient, indicating that literate households are more likely to join cooperatives. Cooperative members are also more likely to have access to credit as the effect of credit access is positive and statistically significant. In addition, Table 2 presents results on the relevance of our instruments. The coefficients on both mobile phone coverage and the presence of private cassava processors are statistically significant at 1%, suggesting the relevance of the instruments.

**Table 2:** Determinants of access to extension and membership of cooperatives.

Variables	Access to extension	Cooperative membership
Household size	0.008	0.029**
	(0.015)	(0.015)
Age	0.029*	−0.003
	(0.015)	(0.015)
Ageˆ2	−0.000	0.000
	(0.000)	(0.000)
Education	0.018**	0.030***
	(0.007)	(0.007)
Marital status	0.044	0.063
	(0.133)	(0.123)
Sex	0.162	−0.139
	(0.134)	(0.125)
TLU	0.002	0.006*
	(0.003)	(0.003)
Land size	−0.023**	0.023**
	(0.010)	(0.010)
Access to credit	0.317***	0.354***
	(0.083)	(0.073)
Distance from fertilizer dealer	0.007**	−0.002
	(0.003)	(0.003)
Use of chemical fertilizer	0.082	0.064
	(0.075)	(0.071)
Distance from herbicide dealer	−0.006*	0.001
	(0.003)	(0.004)
Mobile phone coverage	1.461***	
	(0.158)	
Presence of private cassava processor		0.516***
		(0.080)
*N*	2190	2190

Note: Standard errors clustered at enumeration level are reported in parentheses. *, **, and *** represent statistical significance at the 10%, 5%, and 1% levels, respectively. Controls included in the regression but not reported here include: village dummies, and dummies for road qualities.

### 4.2 Effects on adoption and welfare outcomes

Table 3 reports treatment effect estimates for extension access and cooperative membership using alternative estimation techniques. Columns 1 and 2 present treatment effects of extension access based on PSM and IPWRA specifications. The third column, which is our preferred specification, presents ESR results. Similarly, columns 4 and 5 present the treatment effects of cooperative membership based on PSM and IPWRA specifications while the last column presents the effect of cooperative membership using ESR approach. In general, the reported effects of extension access and cooperative membership are robust across all estimation strategies, showing the important role of extension access and cooperative membership on technology adoption and welfare outcome indicators. In particular, we found that extension access increases the probability of adopting improved cassava varieties by 15.5% using PSM and 20.7% using the IPWRA specifications. Similarly, cooperative membership increases the probability of adopting improved cassava varieties by about 13% both in the PSM and IPWRA specifications. In our ESR model, where we accounted for both observable and unobservable sources of bias, the effect of extension access and cooperative membership is 12.3% and 13.7%, respectively. These results underscore that public investment that aims at improving extension coverage and cooperative membership can have a significant effect on adoption of improved cassava varieties.

**Table 3:** Effect of extension access and cooperative membership on adoption and welfare outcomes.

Variables	Extension access			Cooperative membership		
	PSM	IPWRA	ESR	PSM	IPWRA	ESR
	1	2	3	4	5	6
PPI score	10.45^∗∗∗^	10.26^∗∗∗^	10.74^∗∗∗^	4.57^∗∗∗^	5.98^∗∗∗^	9.01^∗∗∗^
	(1.07)	(0.78)	(0.301)	(1.29)	(0.89)	(0.317)
Adoption	0.155^∗∗∗^	0.207^∗∗∗^	0.123^∗∗∗^	0.134^∗∗∗^	0.133^∗∗∗^	0.137^∗∗∗^
	(0.029)	(0.022)	(0.004)	(0.034)	(0.024)	(0.005)
Log of asset value	0.275^∗∗∗^	0.30^∗∗∗^	0.467^∗∗∗^	0.292^∗∗∗^	0.34^∗∗∗^	0.96^∗∗∗^
	(0.096)	(0.074)	(0.0145)	(0.113)	(0.084)	(0.016)
Food expenditure	0.157^∗∗∗^	0.095^∗∗∗^	0.256^∗∗∗^	0.052	0.077^∗∗^	0.125^∗∗∗^
	(0.051)	(0.034)	(0.013)	(0.054)	(0.037)	(0.009)
N	2090	2090	2090	2090	2090	2090

Robust standard errors are reported in parentheses. *, **, and *** represent statistical significance at the 10%, 5%, and 1% levels, respectively. Village dummies were included but not reported here.

In addition to effects on adoption of improved cassava varieties, Table 3 also presents the effect of extension access and cooperative membership on household welfare (PPI, asset ownership and per-capita food expenditure). Reported results in Table 3 suggest that both extension access and cooperative membership have a positive and statistically significant effect on household welfare indicators. The direction and magnitude of estimated effects are also consistent across all specifications. For instance, the impact of extension access on food consumption ranges from 9.5% in the IPWRA model to 25.6% in the ESR model. These results are consistent with estimates reported by Dercon et al. (2009) for Ethiopia. In addition, the effects of extension access on asset ownership and on PPI score are found to be positive and significant across all specifications. This finding is compelling as it can be used as an additional robustness check for the validity of PPI. The consistent positive and significant effect of extension access on asset ownership, food expenditure and PPI score across all specifications underlines the crucial role that investment in public extension system may play in improving the welfare of rural poor farm households. Coming to the effect of cooperatives on household welfare, results are largely similar to that of extension access. For example, the effect of cooperative membership on PPI score ranges from 4.6 points in the PSM specification to 9.0 points in the ESR estimates. Moreover, we found statistically significant and positive impacts of cooperative membership on asset ownership and food expenditure. These consistent positive impacts of cooperative membership on alternative welfare indicators and adoption imply that addressing output and input market inefficiencies through cooperatives can improve the well-being of rural poor farmers.

Since the reliability of PSM and IPWR results depends on the quality of our matching, we present the extent of overall covariate balancing and the overlap over the common support. The overall covariate balancing test (Table 4) shows that the standardized mean difference for all covariates used in the PSM reduces from 13.6% pre-matching to 4.2% post-matching for extension access. Similarly, the mean standardized bias reduces from 11.5% to 6% for cooperative membership. Moreover, the joint significance of all covariates was never rejected before matching for both extension access and cooperative membership (p>χ2 = 0.000). However, the likelihood ratio tests indicate that the joint significance of all covariates can be rejected after matching (p>χ2 = 0.332 for extension access and p>χ2 = 0.238 for cooperative membership). The low mean standardized bias and joint insignificance of the covariates are indicative of successful balancing of the distribution of covariates between treated and untreated households.

**Table 4:** Propensity score matching quality test.

	Extension access	Cooperative membership
Pseudo R2 before matching	0.044	0.061
Pseudo R2 after matching	0.004	0.01
LRχ^2^ (p-value) before matching	123.87 (p>χ^2^ = 0.000)	142.99 (p>χ^2^ = 0.000)
LRχ^2^ (p-value) after matching	8.17 (p>χ^2^ = 0.698)	15.06 (p>χ^2^ = 0.238)
Mean standardized bias before matching	13.6	11.5
Mean standardized bias after matching	2.8	6.0

**Fig. 1:**
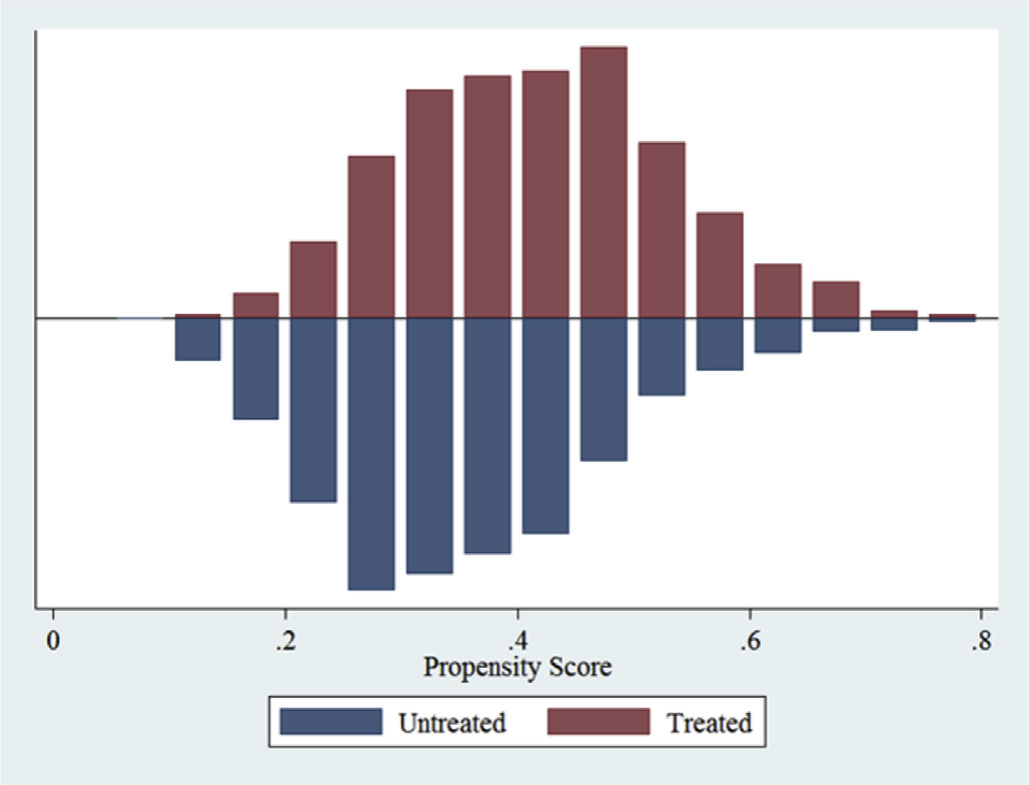
Common support region for extension access.

**Fig. 2:**
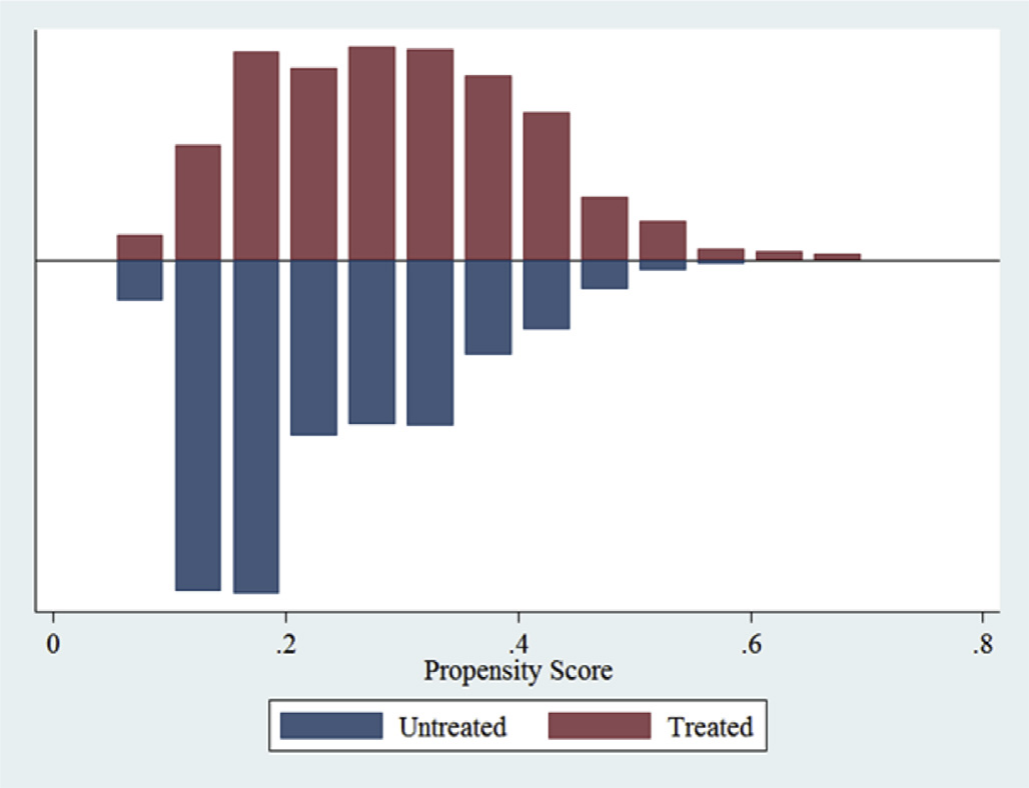
Propensity score distribution and common support for cooperative membership.

In addition, Fig. 1 presents the common support region for extension access and Fig. 2 for cooperative membership. A visual inspection of the distribution of the estimated propensity scores for households with and without treatment indicates that the common support condition is satisfied.

### 4.3 Heterogeneous treatment effects

#### 4.3.1 Heterogeneous treatment effect over propensity scores

**Fig. 3:**
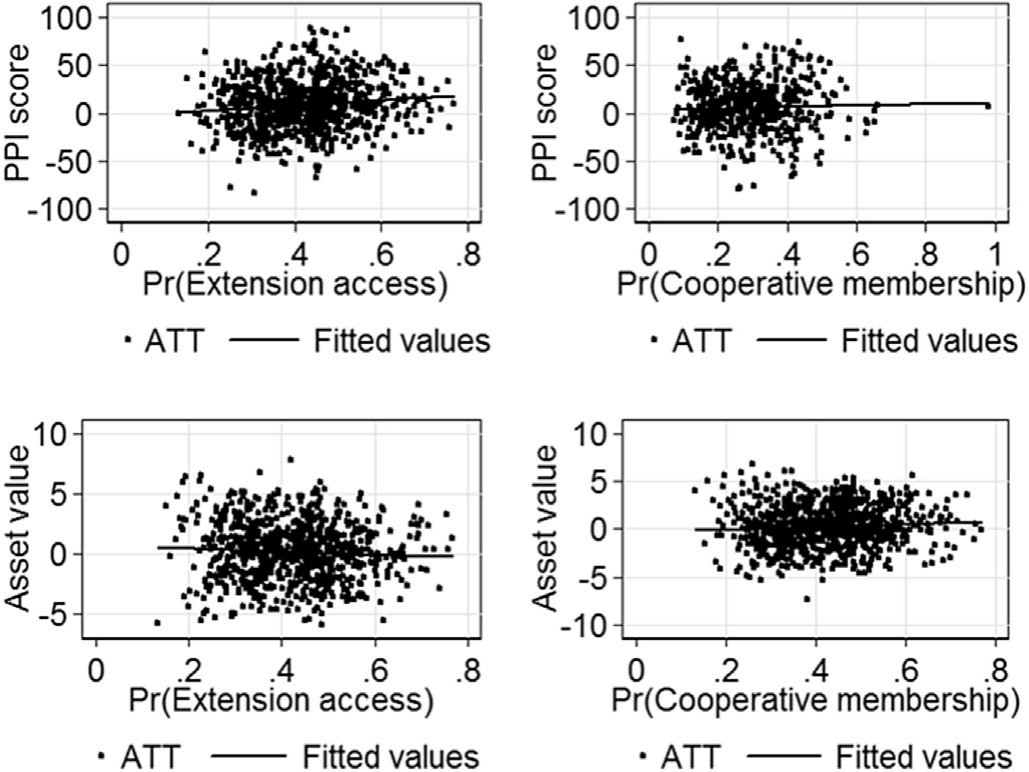
Heterogeneity of treatment effects over the propensity score.

Fig. 3 shows how the average treatment effect on the treated (ATT) of PPI scores and asset values vary over the estimated propensity scores. The results indicate that the ATT on the PPI score varies significantly with the propensity score and that the slope is positive for both extension access and cooperative membership. This result suggests that the effect of extension access on the PPI score is stronger for households with the highest propensity to have extension access. The effect of cooperative membership on the PPI score also increases with the propensity of cooperative membership.

The bottom two figures (Fig. 3) present the estimated average asset values over the estimated propensity score. The results indicate that the ATT on asset value exhibits a significant variation with the propensity score and asset ownership and the probability of extension access have a negative correlation. However, the ATT on asset values exhibits a smaller variation with the propensity score and the correlation is positive.

#### 4.3.2 Heterogeneous effects of extension access over household characteristics

The previous results on the ATT of extension access on outcome indicators highlighted the important role that extension access may play in rural livelihoods. However, the estimated ATTs of extension access on welfare outcome indicators can differ among different sets of farm households. Capturing the differential effect of extension access is therefore important for targeting individual farm households as well as for designing “*best-fit*” extension systems instead of “*one size fits all*” extension approaches. In this section, we present the heterogeneous treatment effect of extension access across various household characteristics in terms of technology adoption and welfare outcomes. Following Verhofstadt and Maertens (2014), we used ATT of individual outcome indicators as a dependent variable in an OLS regression and examined how the estimated ATT may vary with the socio-economic characteristics of farmers. Results are reported in Table 5. (see Table 6)

**Table 5:** Heterogeneous treatment effects of extension.

Variables	Log asset value	Adoption	PPI score
Household size	0.147***	0.000	−2.390***
	(0.038)	(0.011)	(0.413)
Age	−0.003	0.011	0.950**
	(0.039)	(0.011)	(0.420)
Ageˆ2	0.000	−0.000	−0.007*
	(0.000)	(0.000)	(0.004)
Education	0.025	−0.002	1.434***
	(0.017)	(0.005)	(0.182)
Marital status	−0.443	−0.149	−6.277
	(0.371)	(0.107)	(4.015)
sex	0.374	0.011	2.913
	(0.370)	(0.106)	(4.001)
TLU	0.004	−0.008	0.250
	(0.034)	(0.010)	(0.368)
Land size	−0.021	−0.007	0.016
	(0.022)	(0.006)	(0.237)
Access to credit	−0.142	0.016	7.076***
	(0.166)	(0.048)	(1.791)
Distance from market	−0.015	0.008*	−0.088
	(0.015)	(0.004)	(0.166)
N	792	792	792

The dependent variable is the ATT of each respective outcome indicators. Standard errors are reported in parentheses. * Significance at the 10% level. ** Significance at the 5% level. *** Significance at the 1% level.

**Table 6:** Heterogeneous treatment effects of cooperative membership.

Variables	Log asset value	Adoption	PPI score
Household size	0.109**	0.002	−2.394***
	(0.045)	(0.013)	(0.490)
Age	0.116**	0.008	1.217**
	(0.046)	(0.013)	(0.506)
Ageˆ2	−0.001**	−0.000	−0.009**
	(0.000)	(0.000)	(0.005)
Education	0.028	0.006	0.888***
	(0.021)	(0.006)	(0.231)
Marital status	−0.187	0.051	4.377
	(0.435)	(0.125)	(4.729)
sex	−0.048	0.043	−3.324
	(0.421)	(0.121)	(4.582)
TLU	−0.009*	0.002	−0.019
	(0.005)	(0.002)	(0.059)
Land size	−0.014	0.003	−0.127
	(0.026)	(0.008)	(0.286)
Access to credit	−0.159	0.177***	−1.645
	(0.212)	(0.061)	(2.302)
Distance from market	−0.007	0.009*	−0.231
	(0.017)	(0.005)	(0.190)
N	520	520	520

The dependant variable is the ATT of each respective outcome indicators. Standard errors are reported in parentheses. * Significance at the 10% level. ** Significance at the 5% level. *** Significance at the 1% level.

The estimated results show that extension access has heterogeneous effects only to a limited extent. Only household size was significant for the asset ownership indicator, showing that extension access exerts a higher effect for farmers with a larger family size. In terms of adoption of improved cassava varieties, we found no statistically significant differential effects, except for distance from the market. Finally, with regard to the PPI score, we found a statistically significant differential effect of access to extension services among treated households with respect to household size, age, education, and access to credit. These results emphasize the fact that households with better access to credit and where the heads are more educated benefit most from extension services. This implies that expanding rural financial markets and schooling can maximize the potential impact of extension access on household welfare.

#### 4.3.3 Heterogeneous effects of cooperatives over household characteristics

ATT estimates of cooperative membership reported in Table 3 assume homogenous impact of membership for all cooperative members. However, previous studies have shown that the effect of cooperative membership can be heterogeneous within members (Abebaw and Haile, 2013, Verhofstadt and Maertens, 2014, Ma and Abdulai, 2016). Herein, we follow the same procedure to examine the existence of heterogeneous treatment effects of cooperative membership across various household characteristics in terms of technology adoption and welfare outcomes (Table 8). The results show that the impact of cooperative membership on asset ownership is not the same for all members. In particular, we found statistically significant and positive effects of cooperative membership for households with larger family size and for older household heads. The impact of cooperative membership on the likelihood of adoption for members differs based on the level of credit access among cooperative members. In particular, our result suggests that the impact is stronger for households with access to credit. While examining the heterogeneous treatment effect on PPI, we find that cooperative membership has the highest impact for older and more educated members. Interestingly, cooperative membership enhances the welfare (PPI scores) of less wealthy members more strongly than those of wealthier members (using TLU as a proxy for wealth). The emergences of grassroots institutions such as cooperatives can therefore be an important policy instruments for reducing income inequality in rural Nigeria.

## 5 Conclusion

Using unique household level data from rural Nigeria, this paper has examined the potential impacts of institutional arrangements in the form of extension access and cooperative membership on the adoption of improved cassava varieties and household welfare. In doing so, this paper provides both empirical and methodological contributions. Empirically, the paper has addressed the role of institutions in addressing information market imperfections (extension access) and input–output market imperfections (cooperatives). In particular, the analysis undertaken in this paper underscored that both extension access and cooperative membership have heterogenous effects and understanding the potential roles of such heterogeneity is key to improve agricultural productivity. The paper underscored the importance of designing “*best fit*” interventions instead of “*one size fits all*” options by capturing essential heterogeneity among smallholders. Methodologically, the paper goes beyond exploratory studies that establish only “*correlations*”. In particular, this paper uses an endogenous switching regression approach to provide causally interpretable results.

Our main results using the endogenous switching regression approach are summarized as follows: First, we found consistently positive and statistically significant effects of extension access and cooperative membership on technology adoption and household welfare. For instance, extension access enhances the adoption of improved cassava varieties by up to 12.3%. Similarly, the effect of extension access on PPI score reaches as high as 10 points. Our analysis further shows that cooperative membership increases the likelihood of adoption by about 22%. We also found that cooperative membership increases the PPI score by about 9 points.

Secondly, both access to extension and cooperative membership have heterogeneous effects over the propensity score of getting access to extension and joining a cooperative as well as other key household characteristics. In particular, we found a positive relationship between the ATT of welfare outcomes and propensity scores for extension access. This result implies that the effect of extension access is stronger for households with the highest propensity to have extension access. The treatment effect of extension access on asset ownership also depends on some household characteristics, such as formal credit access, education level and household size. We also found differential impacts of extension access with respect to credit access, education, household size, age, and land size for the ATT on PPI score. The consistent positive and significant effect of credit access on these outcome indicators imply that expanding rural financial markets can maximize the potential impact of extension access on household welfare and technology adoption. We also found that the beneficial impact of cooperative membership is stronger for more educated members. In addition, there is differential impact of cooperative membership conditional on household wealth, with stronger and positive effects on relatively poorer members than on wealthier members. A key policy implication of these differential impacts is that the positive impacts of extension and cooperative services on farmers’ productivity and welfare could be reinforced if, for instance, rural financial markets and schools expand.

## Figures and Tables

**Table 7:** Endogenous switching regression estimates on asset ownership

Variables	Asset value		
	Selection	With extension	Without extension
Household size	−0.041	0.491^∗∗∗^	0.649^∗∗∗^
	(0.067)	(0.116)	(0.113)
Age	0.029^∗∗^	0.052^∗∗^	0.049^∗∗^
	(0.015)	(0.0258)	(0.0246)
Ageˆ2	−0.0002	−0.00026	−0.00023
	(0.00013)	(0.00024)	(0.0002)
Education	0.022^∗∗∗^	0.063^∗∗∗^	0.063^∗∗∗^
	(0.0066)	(0.011)	(0.012)
Marital status	0.104	−0.105	−0.094
	(0.14)	(0.244)	(0.229)
Sex	0.047	0.318	−0.425^∗^
	(0.138)	(0.240)	(0.228)
TLU	0.037^∗∗∗^	0.096^∗∗∗^	0.046^∗∗^
	(0.011)	(0.018)	(0.019)
Land size	−0.02^∗∗^	0.0055	0.003
	(0.0097)	(0.014)	(0.0156)
Access to credit	0.234^∗∗∗^	0.147	0.084
	(0.063)	(0.115)	(0.123)
Use of chemical fertilizer	0.055	0.093	0.0009
	(0.07)	(0.119)	(0.0047)
Distance from herbicide market	−0.0066^∗∗^	0.0019	0.005
	(0.00296)	(0.0054)	(0.0048)
Cassava steam market	0.056	0.177	0.051
	(0.076)	(0.121)	(0.131)
Mobile phone coverage	0.213^∗∗∗^		
	(0.096)		
$\sigma_{i}$		0.392^∗∗∗^	0.574^∗∗∗^
		(0.0351)	(0.031)
$\rho_{j}$		0.33^∗∗^	−0.27
		(0.132)	(0.183)
N	2090	815	1275

$\sigma_{i}$ denotes the square-root of the variance of the error terms in the outcome equations. $\rho_{j}$ denotes the correlation coefficient between the error term of the selection equation and the error term of the outcome equations. ∗ Significant at the 10% level; ∗∗ Significant at the 5% level; ∗∗∗ Significant at the 1% level.

**Table 8:** Endogenous switching regression estimates for PPI score

Variables	PPI score		
	Selection	Cooperative members	Non-members
Household size	0.024^∗^	−0.045^∗∗∗^	−0.038^∗∗∗^
	(0.0139)	(0.0078)	(0.0045)
Age	−0.0116	0.014^∗^	0.009^∗∗^
	(0.014)	(0.0079)	(0.004)
Ageˆ2	0.00013	−0.00012^∗^	−0.00007^∗^
	(0.00013)	(0.00007)	(0.00004)
Education	0.026^∗∗∗^	−0.0045	0.022^∗∗∗^
	(0.0063)	(0.0037)	(0.002)
Marital status	0.043	0.0064	−0.08^∗∗^
	(0.128)	(0.072)	(0.039)
Sex	−0.1225	0.027	0.014
	(0.129)	(0.071)	(0.041)
TLU	0.0032	−0.001	0.00009
	(0.003)	(0.0012)	(0.0025)
Land size	0.031^∗∗^	−0.0097^∗∗^	−0.007^∗∗^
	(0.009)	(0.0049)	(0.003)
Access to credit	0.209^∗∗∗^	0.147	0.084
	(0.041)	(0.115)	(0.123)
Distance from herbicide market	0.0008	0.0002	−0.00004
	(0.0027)	(0.0015)	(0.0009)
Cassava steam market	0.048	−0.0043	0.0018
	(0.0049)	(0.0027)	(0.0016)
Presence of private cassava processors	0.188^∗∗∗^		
	(0.049)		
$\sigma_{i}$		−0.617^∗∗∗^	−1.01^∗∗∗^
		(0.044)	(0.018)
$\rho_{j}$		−2.35^∗∗^	−0.135
		(0.149)	(0.095)
N	2090	564	1526

$\sigma_{i}$ denotes the square-root of the variance of the error terms in the outcome equations. $\rho_{j}$ denotes the correlation coefficient between the error term of the selection equation and the error term of the outcome equations. ∗ Significant at the 10% level; ∗∗ Significant at the 5% level; ∗∗∗ Significant at the 1% level.

## Footnotes

1 The ten questions used for constructing the PPI for Nigeria are available online here: http://www.progressoutofpoverty.org/ppi-users.

2 The difference between Eqs. (11), (13) represents ATU.

3 Note that, determinants of extension access and cooperative membership can directly be estimated from the ESR selection equation. However, due to space limitation, we were able to include only some selected results. These selected ESR results are reported in Tables 7 and 8 in the appendix. The full results are available upon request.
